# Supporting Parents of Children With Type 1 Diabetes: Experiment Comparing Message and Delivery Types

**DOI:** 10.2196/41193

**Published:** 2023-02-03

**Authors:** Bree Holtz, Katharine Mitchell

**Affiliations:** 1 Department of Advertising and Public Relations College of Communication Arts and Sciences Michigan State University East Lansing, MI United States

**Keywords:** caregiving, children, development, diabetes, diagnosis, effectiveness, email, intervention, management, social support, stress, support, type 1 diabetes

## Abstract

**Background:**

Type 1 diabetes (T1D) is a chronic condition that typically affects young age group people and is estimated to afflict approximately 154,000 people younger than 20 years in the United States. Since T1D typically impacts children, parents must play an active role in helping their child manage the condition. This creates a substantial burden and responsibility for the parents.

**Objective:**

This pilot study sought to find ways to help parents with children with T1D in coping with stresses related to managing and monitoring their child’s disease by providing informational support, either about parenting a child with T1D or general parenting messages through different channels.

**Methods:**

Parents (N=120) of children with T1D were recruited through an email listserv through local T1D Facebook groups. A total of 102 participants were included in the analysis. We conducted a 2×2 experimental study over an 8-week period to test 2 types of messages (diabetes specific vs general parenting) and the medium in which the messages were delivered (Facebook vs SMS text message). Diabetes behavior, informational support, emotional support, and quality of life were the main outcomes of interest.

**Results:**

The results suggested that the participants in the diabetes message groups showed improvement in diabetes behaviors (*F*_1,99_=3.69; *P*=.05) and were more satisfied with the intervention (*F*_3,98_=4.59; *P*=.005). There were no differences between message and medium groups on informational support, emotional support, or quality of life.

**Conclusions:**

The results of this study demonstrate that the medium—Facebook or SMS text messaging—does not matter for parents’ perceptions of social support or quality of life. The diabetes message group reported higher levels of disease management. Finally, the groups with the diabetes support messages were more satisfied than those who received general parenting messages. The findings provide starting guidance for the development of social support interventions for this population.

## Introduction

Chronic conditions impact nearly 40% of children and adolescents in the United States [1]. Type 1 diabetes (T1D) is a chronic condition that typically affects young age group people. T1D afflicts approximately 154,000 people younger than 20 years in the United States [2,3]. For children younger than 10 years, the prevalence of diagnosis is 19.7 per 100,000/year. For adolescents aged 10-19 years, the prevalence is 18.6 per 100,000/year [4]. T1D costs US $14.9 billion annually in the United States [5,6]. Currently, approximately 75% of adolescents are not achieving the American Diabetes Association’s hemoglobin A_1c_ (HbA_1c_) targets [7-10]. HbA_1c_ is a measure of an individual’s average blood sugar over the past 3 months. These statistics often do not consider the number of parents and caregivers of these children who are also impacted by the burden of disease management. Parents of children with T1D are often the main caregiver for their child [11].

Parents who have a child with T1D have a substantial responsibility in managing their child’s disease. As T1D typically impacts children, parents typically play a very active role in managing the condition [12]. The diagnosis can affect families for the rest of their lives [13]. Daily life with diabetes requires adherence to an extremely complex care plan, involving multiple doctor visits, extensive health education, daily blood glucose monitoring, insulin injections, and the careful monitoring of diet and physical activity. The diagnosis can forever change the psychological dynamics of the family. Parents of children with T1D often struggle with stress, depression, and anxiety over the care of their child. They may also experience increases in family conflict and feelings of burnout [13].

Many parents report feeling overwhelmed by all the disease management information accompanying a diagnosis of T1D, leading to feelings of stress and isolation, which can lead to worse health outcomes for the parents. Social support, which is defined here as the perception of being part of a supportive social network [14], has often been cited as a way to help parents cope with this stress, improve overall health status, and act as a buffer for various effects of stress [12]. Parents with a child who has a chronic condition may feel higher levels of stress if they perceive that they do not have the required knowledge or support systems needed to cope with the demands of caring for a child with a life-long condition, like T1D. Information communication technologies (ICTs; eg, social media, online forums, SMS text messages) are one way to help reduce feelings of stress and isolation by improving social support [15]. These types of technologies allow people to engage in supportive communities that transcend time constraints and geography. Previous research suggests that parents of children with T1D gain multiple benefits by using ICTs for support and information [16,17].

This pilot study sought to understand if the types of parenting messages (diabetes specific or general) and the ICT medium in which the messages were sent (Facebook or SMS text message) impacted the parents’ perceptions in multiple dimensions. Specifically, social support, quality of life, management of their child’s T1D, and satisfaction with the intervention were explored. This pilot study is a first step in developing supportive programming for parents with a child with T1D. Based on this purpose, the following hypotheses and research question were developed for this study:

- Hypothesis 1: The Facebook group, regardless of message type, will have higher perceptions of social support and quality of life.
- Hypothesis 2: Both groups that received diabetes messages will improve on diabetes management.
- Research question 1: How will the groups differ in satisfaction when considering both channel and message?

## Methods

### Study Design

This pre-post test survey pilot study was a 2 (SMS text messaging vs Facebook) × 2 (general parenting messages vs diabetes-specific messages) between-subjects factorial design. Participants (n=120) were randomly assigned to 1 of the 4 conditions over an 8-week period. Participants were recruited through a listserv of parents with children with T1D and through local T1D Facebook groups.

### Participants and Procedures

We recruited 120 participants within 7 days. To be sure that the participants met our inclusion criteria, the participants answered the following 3 screening questions to be included in the study: (1) have a child (any age) living at home with T1D, (2) have a mobile phone that can send and receive SMS text messages, and (3) have a Facebook account. Then, participants were randomized, using a random number generator, into 1 of the 4 experimental groups: (1) Facebook and general messages, (2) Facebook and diabetes messages, (3) SMS text messaging and general messages, and (4) SMS text messaging and diabetes messages. Randomization was done upon passing the screening, before the start of the study, because the consent form was included within the pretest survey and the conditions needed to be determined beforehand for participants to receive the appropriate survey link. Each of the survey links contained the same questionnaire but enabled the samples to be easily separated. The consent form varied slightly between the groups to reflect the difference in the procedures (ie, which channel they used for communication). On day 1 of the study, participants were emailed a link to the survey that included the consent form, and once completed, they continued to the pretest survey. Participants were given 5 days to complete the pretest survey. Completion of the pretest survey was tracked by the researchers. During the intervention period, participants were sent approximately 3 messages per week (a total of 23 messages) for 8 weeks during the fall of 2019 (either via SMS text message or posted to the Facebook group). For this study, we developed 2 private Facebook groups (Diabetes and General) and enrolled those who were randomized into the appropriate group. All messages (Facebook and SMS text messages) were scheduled to go out on the same day and time. The participants could respond to the SMS text messages and Facebook posts through comments (see Textbox 1 for examples of the messages).

**Figure 1:**
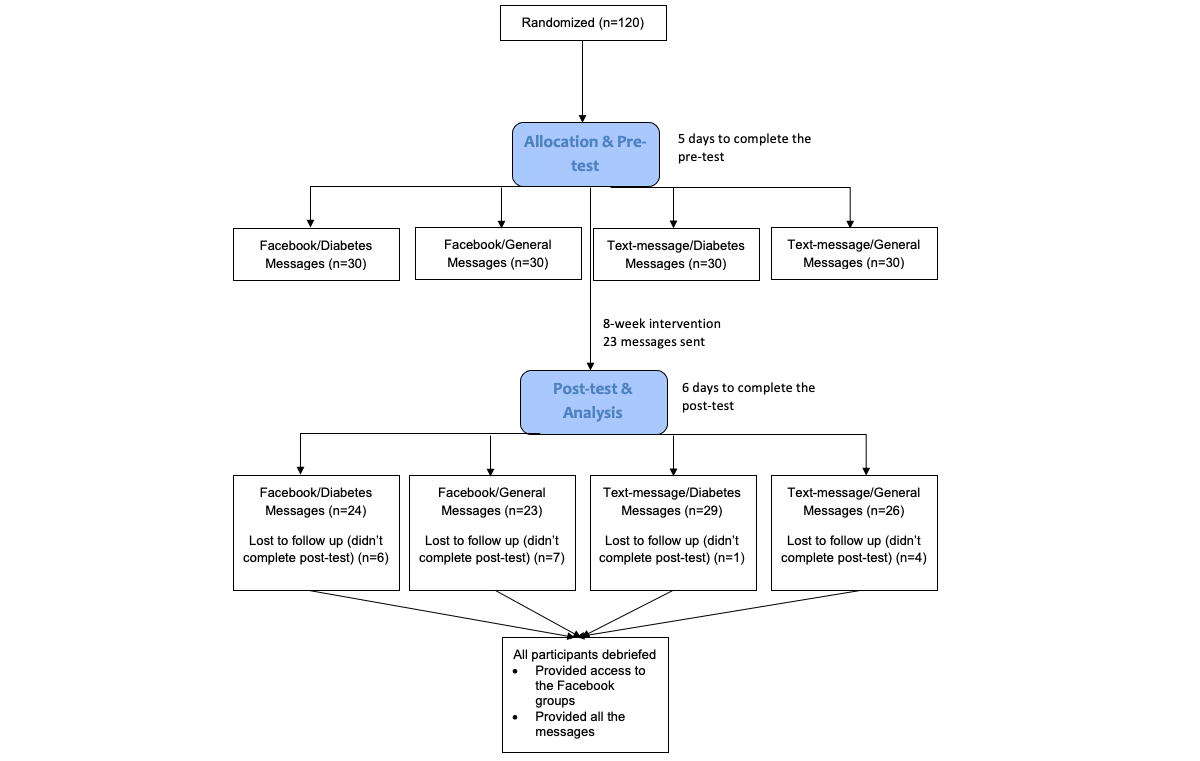
Enrollment and randomization.

After the 8-week duration, participants were asked to take an online posttest survey. Participants were given 6 days to complete the posttest survey. Completion of the posttest survey was tracked by the researchers and a debrief email was sent once the survey was complete. The debrief disclosed the full purpose of the study, provided a PDF document of all the messages (general and diabetes specific), and provided links to access both Facebook groups (Figure 1).

**Message examples.**

**Type 1 diabetes related**

- Being a parent of a child with type 1 diabetes is a 24/7/365 job. Be sure to be kind to yourself. You’re doing great!
- It can be hard to find a reliable person to help take care of your child. For more details, the reader can refer to [18].
- Let your child know when he/she is doing a good job managing his/her diabetes.

**General parenting**

- Try a healthier way to handle stress this week: Go for a walk, read a book, listen to music, get enough sleep, or do a favorite hobby. By actively coping with stress, you can prevent running out of fuel throughout the week.
- By taking care of your own needs, you will be better able to respond and help your child.
- Next time you get in a disagreement with your child, take three deep breaths and try responding in a more friendly tone. Fighting anger with anger usually leads to a lose-lose situation for you and your child.

### Measures

The pre- and posttest surveys were nearly identical. The only differences were that the pretest administered demographic questions and only the posttest administered the satisfaction questions. All of the scales used for this study are validated measures. Informational support was measured using the 6-item PROMIS Item Bank v2.0–Informational Support–Short Form 6a (*α*=.95), which is on a 5-point scale from “Never” to Always.” Higher scores indicated greater feelings of support. Emotional support was measured using the 8-item PROMIS Item Bank v2.0–Emotional Support–Short Form 8a (*α*=.96) that uses a 5-point scale from “Never” to “Always.” Higher scores indicated greater feelings of support [19]. To measure the participants’ quality of life, a modified version (to indicate T1D) of The Pediatric Caregiver’s Quality of Life Questionnaire (*α*=.88) was used [20]. This scale has 7 response options ranging from “All of the time” to “None of the time.” A lower score indicated worse quality of life. The surveys included 7 items of The Diabetes Behavior Rating Scale (*α*=.68), which assesses how much the parent is following the recommended treatment plan for their adolescent [21]. This scale has 5 responses categories ranging from “Never” to “Always.” A higher score indicates a greater level of adherence. Satisfaction of the intervention was also measured using 4 items on a 7-point scale from “Extremely unsatisfied” to “Extremely satisfied” (*α*=.89). Higher scores indicated greater satisfaction.

### Statistical Analysis

Descriptive statistics were used to profile the sociodemographic characteristics of the parents. ANOVA was used to assess the differences between groups. Furthermore, post hoc comparisons were conducted using Tukey HSD tests.

### Ethics Approval

This study was approved by the Institutional Review Board of Michigan State University (MSU Study ID: STUDY0000274). The institutional review board of the university approved the study procedure and protocols and determined this an exempt study under the Flexibility Initiative Exemption Category 98, which is research involving benign interventions in conjunction with the collection of data from adult subjects that are considered to be minimal risk. All participants agreed to participate through the informed consent process in which they were informed of the study tasks, risks and benefits of participating, and the voluntary nature of their participation. All identifying information about the participants was kept separate from their survey responses, and all data were completely deidentified. Once the pretest survey was completed, participants received a US $5 gift card to a national coffee chain. Once the posttest surveys were completed, they received a second US $5 gift card to a national coffee chain.

## Results

The analysis includes participants that completed both the pre- and posttest surveys (n=102). See Table 1 for participant demographics.

Hypothesis 1 proposed that the Facebook group, regardless of message type, would have higher perceptions of social support and quality of life. ANOVA results suggest that there was not a significant effect of group type (Facebook vs SMS text messaging) on informational support (*F*_1,100_=0.189; *P*=.66), emotional support (*F*_1,100_=0.019; *P*=.89), or quality of life (*F*_1,98_=0.65; *P*=.42). This suggests that the channel through which the participants received the messages did not influence their perception of social support or quality of life. Means for all groups can be found in Table 2. Hypothesis 1 was therefore not supported.

Hypothesis 2 proposed that both groups that received diabetes messages would improve on diabetes management. Results suggest that there was a significant effect of diabetes messages compared to general messages regarding diabetes adherence (*F*_1,99_=3.69; *P*=.05). The groups that received the diabetes messages reported more diabetes adherence behaviors. Therefore, hypothesis 2 is supported.

Research question 1 aimed to explore how the general parenting messaging groups differed from the diabetes messaging groups. Results suggest that there was a significant difference between groups on satisfaction (*F*_3,98_=4.59; *P*=.005). Therefore, a post hoc comparison using a Tukey honestly significant difference test was conducted and indicated that the Facebook diabetes messaging group was significantly more satisfied than the Facebook general messaging group (*P*=.009). Additionally, the SMS text messaging diabetes messages group was significantly more satisfied than the Facebook diabetes messages group (*P*=.01). There were no other differences in satisfaction between groups.

**Table 1:** Participant demographics.

Variable		Total (n=102), n (%)	Group, n (%)			
			Facebook and general (N=23)	Facebook and diabetes (N=24)	SMS text messaging and general (N=26)	SMS text messaging and diabetes (N=29)
**Relationship to child**						
	Biological mother	94 (92.2)	21 (91.3)	24 (100)	24 (92.3)	25 (86.2)
	Biological father	7 (6.9)	2 (8.7)	—^a^	2 (7.7)	3 (10.3)
	Adoptive mother	1 (1)	—	—	—	1 (3.4)
**Age (years)**						
	25-29	8 (7.8)	2 (8.7)	—	2 (7.7)	4 (13.8)
	30-39	35 (34.4)	9 (39)	9 (37.5)	11 (42.3)	6 (20.7)
	40-49	51 (50)	10 (43.7)	13 (54.1)	12 (46.2)	16 (55.2)
	50-59	7 (6.8)	1 (4.3)	2 (8.4)	1 (3.8)	3 (10.3)
	≥60	1 (1)	1 (4.3)	—	—	—
**Age of child (years)**						
	≤5	4 (4.1)	1 (4.5)	2 (9.1)	1 (4)	—
	6-10	43 (44.4)	8 (36.4)	11 (50)	11 (44)	13 (46.4)
	11-15	38 (39.1)	9 (40.9)	7 (31.8)	9 (36)	13 (46.4)
	16-18	10 (10.3)	3 (13.7)	2 (9.1)	3 (12)	2 (7.2)
	≥19	2 (2.1)	1 (4.5)	—	1 (4)	—
**Length of diagnosis**						
	<6 months	7 (7.5)	2 (9.5)	1 (4.8)	2 (8.7)	2 (7.1)
	6 months to <1 year	9 (9.7)	2 (9.5)	2 (9.5)	2 (8.7)	3 (10.7)
	1 year to <5 years	51 (54.8)	11 (52.4)	15 (71.4)	12 (52.2)	13 (46.4)
	≥5 years	26 (28)	6 (28.6)	3 (14.3)	7 (30.4)	10 (35.7)
**Child treatment^b^**						
	Pump	69 (67.6)	15 (65.2)	16 (66.7)	19 (73.1)	19 (65.5)
	Continuous glucose monitor	87 (85.3)	19 (82.6)	21 (87.5)	22 (84.6)	25 (86.2)
	Injections	32 (31.4)	9 (39.1)	7 (29.2)	6 (23.1)	10 (34.5)
**Education**						
	No schooling completed	1 (1)	—	1 (4.2)	—	—
	Some high school, no diploma	1 (1)	—	—	—	1 (3.4)
	High school diploma	6 (5.9)	2 (8.7)	—	2 (7.7)	2 (6.9)
	Some college credit, no degree	20 (19.6)	6 (26.1)	5 (20.8)	4 (15.4)	5 (17.2)
	Trade/technical/vocational training	5 (4.9)	2 (8.7)	2 (8.3)	1 (3.8)	—
	Associate degree	13 (12.7)	4 (17.4)	1 (4.2)	6 (23.1)	2 (6.9)
	Bachelor’s degree	37 (36.3)	5 (21.7)	9 (37.5)	10 (38.5)	13 (44.8)
	Master’s degree	15 (14.7)	3 (13)	5 (20.8)	3 (11.5)	4 (13.8)
	Doctorate degree	1 (1)	—	1 (4.2)	—	—
	Professional degree	3 (2.9)	1 (4.3)	—	—	2 (6.9)
**Marital status**						
	Single, never married	4 (3.9)	2 (8.7)	1 (4.2)	—	1 (3.4)
	Married or domestic partnership	88 (86.3)	18 (78.3)	22 (91.7)	22 (84.6)	26 (89.7)
	Widowed	1 (1)	1 (4.3)	—	—	—
	Divorced	8 (7.8)	1 (8.7)	—	4 (15.4)	2 (6.9)
	Separated	1 (1)	—	1 (4.2)	—	—
**Employment status**						
	Employed for wages	66 (65.3)	16 (72.7)	16 (66.7)	13 (50)	21 (72.4)
	Self-employed	5 (5)	1 (4.5)	1 (4.2)	1 (3.8)	2 (6.9)
	Out of work and looking	2 (2)	—	—	1 (3.8)	1 (3.4)
	Out of work but not looking	1 (1)	—	—	—	1 (3.4)
	A homemaker	23 (22.8)	5 (22.7)	6 (25)	9 (34.6)	3 (10.3)
	A student	2 (2)	—	—	2 (7.7)	—
	Military	1 (1)	—	—	—	1 (3.4)
	Unable to work	1 (1)	—	1 (4.2)	—	—
**Income (US $)**						
	Less than 25,000	5 (5.1)	1 (4.3)	1 (4.2)	—	3 (11.1)
	25,000-34,999	4 (4.1)	2 (8.7)	—	1 (4.2)	1 (3.7)
	35,000-49,999	10 (19.2)	4 (17.4)	1 (4.2)	2 (8.3)	3 (11.1)
	50,000-74,999	21 (21.4)	5 (21.7)	4 (16.7)	7 (29.2)	5 (18.5)
	75,000-99,999	19 (19.4)	4 (17.4)	3 (12.5)	7 (29.2)	5 (18.5)
	100,000-149,999	16 (16.3)	3 (13)	5 (20.8)	5 (20.8)	3 (11.1)
	≥150,000	23 (23.5)	4 (17.4)	10 (41.7)	2 (8.3)	7 (25.9)
**Race**						
	White	89 (90.8)	21 (91.3)	20 (90.9)	23 (92)	25 (86.2)
	Hispanic	5 (5.1)	2 (8.7)	1 (4.5)	2 (8)	1 (3.4)
	Black	1 (1)	—	—	—	1 (3.4)
	Native American or American Indian	2 (2)	—	—	—	2 (6.9)
	Asian	1 (1)	—	1 (4.5)	—	—

^a^Not applicable.

^b^Child treatment categories may not equal 100% as respondents were asked to check all that apply.

**Table 2:** Comparison of all groups’ pre- and posttest means and standard deviations.

Group		All FB^a^ (n=47), mean (SD)	All SMS text messaging (n=55), mean (SD)	All general (n=49), mean (SD)	All diabetes (n=53), mean (SD)	FB and general (n=23), mean (SD)	FB and diabetes (n=24), mean (SD)	SMS text messaging and general (n=26), mean (SD)	SMS text messaging and diabetes (n=29), mean (SD)
**Pretest**									
	Informational support	3.83 (1.00)	3.72 (0.98)	3.69 (1.07)	3.84 (0.91)	3.74 (1.18)	3.92 (0.83)	3.65 (0.99)	3.77 (0.99)
	Emotional support	4.16 (0.88)	3.88 (1.02)	4.02 (1.02)	4.00 (0.92)	4.17 (0.91)	4.16 (0.88)	3.87 (1.11)	3.88 (0.94)
	Quality of life	4.44 (1.02)	4.50 (1.11)	4.42 (1.14)	4.5 (1.00)	4.43 (1.06)	4.46 (1.00)	4.40 (1.22)	4.59 (1.01)
	Diabetes Behavior Rating Scale	3.66 (0.62)	3.74 (0.73)	3.61 (0.65)	3.78 (0.71)	3.59 (0.59)	3.71 (0.66)	3.63 (0.70)	3.84 (0.75)
	Satisfaction	—^b^	—	—	—	—	—	—	—
**Posttest**									
	Informational support	3.91 (0.94)	3.97 (0.83)	3.92 (0.92)	3.97 (0.86)	3.90 (0.99)	3.91 (0.93)	3.92 (0.87)	4.02 (0.81)
	Emotional support	4.00 (0.93)	4.03 (0.98)	4.02 (0.93)	4.01 (0.98)	4.04 (0.93)	3.96 (0.95)	4.00 (0.95)	4.05 (1.02)
	Quality of life	4.43 (1.25)	4.70 (1.39)	4.48 (1.45)	4.67 (1.21)	4.42 (1.42)	4.44 (1.11)	4.52 (1.50)	4.85 (1.28)
	Diabetes Behavior Rating Scale	3.81 (0.63)	3.75 (0.73)	3.63 (0.63)	3.91 (0.74)^c^	3.67 (0.44)	3.93 (0.75)	3.59 (0.76)	3.89 (0.74)
	Satisfaction	5.04 (1.25)	5.34 (1.41)	4.78 (1.24)	5.60 (1.32)	4.43 (1.08)	5.63 (1.14)^c^	5.08 (1.31)	5.57 (1.48)^d^

^a^FB: Facebook.

^b^Not applicable.

^c^*P*≤.05.

^d^*P*≤.01.

## Discussion

Caring for a child or adolescent with T1D can be incredibly stressful for parents. This study was designed to determine if the type of message or the medium was important for parent support. This study found that there was no difference in perceived support or quality of life between the Facebook groups and the SMS text messaging groups, suggesting that the channel of the support messages does not matter. The data also confirm that receiving messages regarding diabetes management did improve perceptions of diabetes adherence. Finally, we found that the targeted messages regarding diabetes parenting were more well liked than the general parenting messages.

Past work has demonstrated that Facebook private groups can provide an online community of peers facing similar circumstances [16]. This study demonstrated that people could feel supported through SMS text messages as well. It was the type of message that was the key to the differences. Parents of children with T1D might not feel that they get enough information specific to parenting a child with a chronic illness. This can be due to a variety of factors, including not knowing any other parents with a child with T1D, always feeling a need to have more information, and not feeling empowered to ask for support [22-26].

This work is important as there is little work regarding providing support messages through ICTs to the parents/caregivers of children or adolescents with a chronic condition [27]. Much of the work is centered on the individual with a chronic illness [28-32]. This pilot study may help others when considering how to design and develop a support group for informal caregivers of people with chronic illnesses. Past studies have shown that adding personalization (ie, name and gender) to the messages may improve perceptions of support of the parents, and it should be considered for future studies [33-37]. This also hints to why the diabetes-specific message had a stronger effect when comparing it to the general parenting message. The findings of this study suggest that the medium in which the messages are delivered did not matter. This indicates that providing options for individuals based on their own preferences will likely have more positive outcomes and likely reduce barriers for engagement. Past research has also noted that parents with high internet self-efficacy were comfortable using Facebook [38]. In another study, parents found SMS text messaging to be desirable [37], thus allowing parents to choose that the modality can improve adoption and long-term use. Parents are likely to be able to find a variety of general parenting support, but for caregivers, diabetes-specific support is likely to be more effective in improving diabetes management skills. Additionally, the diabetes-messages groups were more satisfied with the intervention than the general parenting groups, strengthening this conclusion.

As with any study, this work also has some limitations. One of which includes the parent population, which was mostly White and women. Future work should ensure a more representative group of parents to understand how that might impact these perceptions. Additionally, all of the surveys were self-reported, and there are known biases when using these types of measures. This study explains social support provided via Facebook or SMS text messaging to informal caregivers, particularly parents of a child with a chronic illness. Additionally, the age of the child was not part of the inclusion criteria, while the vast majority (n=81, 83.5%) had children between the age of 6-15 years, and the needs of these parents are most likely different in terms of the messages needed. The findings provide guidance for the development of social support interventions for this population.

## Data Availability

BH is the guarantor of this work and, as such, had full access to all the data in the study and takes responsibility for the integrity of the data and the accuracy of the data analysis.

## Abbreviations

HbA_1c_: hemoglobin A_1c_
ICT: information and communication technology
T1D: type 1 diabetes
